# Perinatal health of refugee and asylum-seeking women in Sweden 2014–17: a register-based cohort study

**DOI:** 10.1093/eurpub/ckz120

**Published:** 2019-07-04

**Authors:** Can Liu, Mia Ahlberg, Anders Hjern, Olof Stephansson

**Affiliations:** 1 Clinical Epidemiology Division, Department of Medicine, Karolinska Institutet, Stockholm, Sweden; 2 Department of Public Health Sciences, Centre for Health Equity Studies, Karolinska Institutet/Stockholm University, Stockholm, Sweden; 3 Department of Women’s and Children’s Health, Division of Obstetrics and Gynecology, Karolinska University Hospital and Institutet, Stockholm, Sweden

## Abstract

**Background:**

An increasing number of migrants have fled armed conflict, persecution and deteriorating living conditions, many of whom have also endured risky migration journeys to reach Europe. Despite this, little is known about the perinatal health of migrant women who are particularly vulnerable, such as refugees, asylum-seekers, and undocumented migrants, and their access to perinatal care in the host country.

**Methods:**

Using the Swedish Pregnancy Register, we analyzed indicators of perinatal health and health care usage in 31 897 migrant women from the top five refugee countries of origin between 2014 and 2017. We also compared them to native-born Swedish women.

**Results:**

Compared to Swedish-born women, migrant women from Syria, Iraq, Somali, Eritrea and Afghanistan had higher risks of poor self-rated health, gestational diabetes, stillbirth and infants with low birthweight. Within the migrant population, asylum-seekers and undocumented migrants had a higher risk of poor maternal self-rated health than refugee women with residency, with an adjusted risk ratio (RR) of 1.84 and 95% confidence interval (95% CI) of 1.72–1.97. They also had a higher risk of preterm birth (RR 1.47, 95% CI 1.21–1.79), inadequate antenatal care (RR 2.56, 95% CI 2.27–2.89) and missed postpartum care visits (RR 1.15, 95% CI 1.10–1.22).

**Conclusion:**

Refugee, asylum-seeking and undocumented migrant women were vulnerable during pregnancy and childbirth. Living without residence permits negatively affected self-rated health, pregnancy and birth outcomes in asylum-seekers and undocumented migrants. Pregnant migrant women’s special needs should be addressed by those involved in the asylum reception process and by health care providers.

## Introduction

Tens of millions of refugees have been trying to find security outside their country of origin because of armed conflicts, persecution and poverty. The overwhelming majority of the world’s refugees resettle in low-income and middle-income countries in Africa and Asia,^1^ but in recent years an increasing number have found their way to Europe, making migration a key issue in many countries in the European Union (EU).^2^ In 2015, Sweden received the second highest number of asylum applications in the EU.^3^

Refugee women are a vulnerable group at risk for sexual and gender-based violence before and during migration, and even after arrival in the destination country,^4^^,^^5^ which can result in mental distress and adverse reproductive health.^6–8^ Although the sexual and reproductive health rights and access to care of refugee women are protected by international conventions^9^ and a binding EU directive,^10^ many refugee women face barriers to accessing the care to which they are entitled.^11–13^

In Sweden, all women are guaranteed by law to have equal access to critical care during pregnancy, birth and the postpartum period, regardless of immigration status.^14^^,^^15^ The Swedish Pregnancy Register^16^ provides comprehensive population-level perinatal information, including asylum-seekers and undocumented migrants who are not systematically recorded in other national registries. Furthermore, antenatal, delivery and postnatal care information are integrated, providing a unique opportunity to investigate perinatal health and health care. With this rich information, we examined the perinatal health of the female refugee population during 2014–17, focusing on residency status as a determinant of perinatal health among migrants.

## Methods

### Setting

Asylum-seekers apply for refugee status upon arrival to the host nation. Refugees with an approved refugee status in Sweden have permanent or extendable temporary residence lasting at least 13 months^17^ and the right to universal medical care with a unique personal identification number.^18^

Perinatal care is free for all women living in Sweden, regardless of their residency status. A temporary number is used for perinatal care for women without a permanent personal identification number. Approximately 99% of the births in Sweden take place in 46 delivery hospitals, each with their own catchment area and local antenatal units.^16^ Women are recommended to have at least nine antenatal visits to midwives during pregnancy and an ultrasound screening before 23 gestational weeks.^19^ The midwives book the delivery hospital, then the woman calls the hospital herself for instructions when she goes into labour. The woman has one postpartum visit to midwives in 6–12 weeks postpartum and the family also receives postnatal home visits and well-baby clinic check-ups.^20^

### Data

The new national Swedish Pregnancy Register provides comprehensive information on each pregnancy, collected from the first antenatal health care visit to the postpartum visit. Electronic medical records are linked with data collected by midwives at the first antenatal visit and the postpartum visit, including the mother’s country of birth, education and self-rated health.^16^ Since 2014, the Swedish Pregnancy Register has included information on 92% of all births.^16^

**Figure 1:**
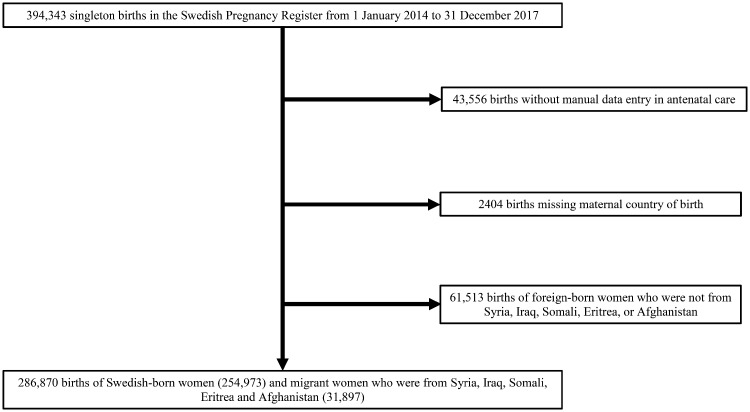
Flowchart of the study population

All 394 343 singleton births registered in the Swedish Pregnancy Register in 2014–17 were eligible for study inclusion (figure 1). We excluded 45 960 births with missing data on antenatal care or the mother’s country of birth, 7356 of which were born to women without personal identification numbers. The final cohort comprised 348 383 (88.3%) of the births registered during the study period, including births of women from Syria, Iraq, Somali, Eritrea and Afghanistan (*N* = 286 870) which comprised the top five countries of origin for asylum-seekers in Sweden from 2014 to 2016.

### Migration status

We used maternal country of birth to identify the migrant population, including refugees, asylum-seekers and undocumented migrants. Women from the top five asylum-seeker countries of origin who had a unique personal identification number were classified as refugees. Women who only had a temporary number in the register were classified in the group of asylum-seekers and undocumented refugees.

### Maternal and neonatal behavioural and health indicators

Information on parity, education level, smoking and alcohol consumption and self-rated health prior to pregnancy were collected at the first antenatal care visit. Self-reported health was rated as very good, good, neither poor nor good, poor, very poor and do not know. Women who provided any answer apart from very good or good were classified as having poor self-rated health prior to pregnancy. Maternal body mass index (BMI) was derived from body height and weight at the first antenatal care visit and maternal age at delivery was retrieved from the medical records.

We examined the relevant International Classification of Diseases, Tenth Edition codes. Pre-eclampsia (codes O14 and O15), gestational diabetes (code O24) and III–IV degree anal sphincter tears (codes O702, O703 and O709), which involves the anal sphincter and can be associated with maternal morbidity such as faecal incontinence, were retrieved from maternal diagnosis in antenatal care and at discharge from the delivery hospital. Severe postpartum haemorrhage was defined as having a blood loss of more than 1000 ml, according to the medical records. Maternal death was defined with code O95, O96 or O97 or an infant diagnosis of P016 indicating maternal death.

Stillbirths were directly retrieved from the medical records. Gestational age at birth was estimated from the date of *in vitro* fertilization, when applicable, ultrasound scans or the last menstrual period when an ultrasound estimation was not available. Preterm birth before 37 gestational weeks was primarily based on gestational age using the same information. Preterm births that were only defined by the estimated gestational age on ultrasound were also compared to avoid differences due to the method of gestational age estimation. Small for gestational age was defined as a birthweight below two standard deviations for gestational age, according to the sex-specific Swedish standard curve of foetal growth.^21^ We hypothesized that migrant babies would have a higher chance of being small for gestational age than native Swedes because of poorer maternal health and nutritional status. Therefore, we compared the risk of having a birthweight below 2500 g and the mean birthweight of term-born infants (39–41 gestational weeks) to triangulate the effect on foetal growth. The Apgar scores 5 min after birth were dichotomized as up to six points or seven points or more.

### Perinatal care

The number of antenatal care visits and ultrasound exams were retrieved from medical records. Having fewer than nine visits was regarded as below the recommended number of antenatal care visits. Having no ultrasound screening or ultrasound estimated gestational age was coded as having no ultrasound screening. We calculated the expected number of antenatal visits based on the Swedish antenatal care programme.^19^ Having less than 50% of the expected number of visits was defined as having inadequate antenatal care.

Medical records also provided information on the use of professional translators during antenatal care, abnormal cardiotocography (CTG) tracing on admission to the delivery ward, the mode of delivery and the date of the postpartum visit. Mode of delivery was split into four categories: non-instrumental or instrumental vaginal deliveries and planned or unplanned Caesarean sections. If there was no date recorded for the postpartum visit, it was defined as a missing visit. The way postpartum visits were organized may have differed between different maternity units, as some book them during late pregnancy, while others ask the woman to call after birth and book the visit herself, which may be difficult for refugees who are unfamiliar with the health system or face language barriers. Unfortunately, the data did not allow us to differentiate between the type of maternity units, which is not associated with individual characteristics or quality of care to our knowledge. Having no recorded postpartum visit was classified as a less adequate use of postpartum care.

### Statistical analysis

Logistic regression models were used to analyze binary outcomes and multinomial logistic regression models were used for categorical outcomes, such as mode of delivery. Adjusted risk ratios (RRs) and risk differences (RDs) were then calculated. The confidence intervals were estimated by delta-method standard errors.^22^ A linear regression model was used on the birthweight of born at term (39–41 weeks).

We first described the perinatal health of all migrants in this study, by comparing their perinatal health outcomes to the Swedish-born women. We reported the observed risks and mean birthweights of term births in both groups along with the relative risks and RDs between them, adjusting for maternal age and parity in quadratic forms.

We further estimated the effect of not having residency on perinatal health by comparing the asylum-seekers and undocumented migrants to refugees. Maternal education, age and parity in quadratic forms, calendar year of delivery and country of origin were adjusted in all models. Additional adjustments for maternal BMI categories and heights, in continuous forms, were made to analyze the risk of adverse maternal and foetal outcomes measured during delivery.

### Sensitivity analysis

Due to large mass migration during a relatively short period, a sensitivity analysis was conducted for Syrian women, to avoid confounding of the measures by duration of residence. We also analysed the outcomes of births with no information on antenatal care and no personal identification numbers, who might have presented only at delivery care (*N* = 7356), restricting the analysis to outcomes measured during or shortly after delivery. Due to missed information on maternal country of birth, they were not included in our study population. Undocumented migrants might be over-represented in this population.

All data management was performed using SAS^®^ version 9.4 (SAS Institute, Cary, NC, USA) and all data analyses with STATA^®^ version IC 14 (64 bit) (StataCorp LLC, College Station, TX, USA).

## Results

We evaluated records of 286 870 women who gave singleton births, 88.9% of whom were Swedish-born, 10.4% who were refugees and 0.7% who were asylum-seekers and undocumented migrants. Table 1 presents the maternal characteristics of migrant and Swedish-born women. The migrant group included more teenage mothers than the Swedish group, as well as more multiparous women, fewer women with a normal BMI, fewer women with risky alcohol behaviour and more women with a delayed first antenatal care visit.

**Table 1:** Description of maternal characteristics and time of the first antenatal care by migration status (*N* = 286 870)

		Asylum-seekers and undocumented migrants		Refugee women		Swedish-born women	
		*N* = 1983		*N* = 29 914		*N* = 254 973	
		*n*	(col %)	*n*	(col %)	*n*	(col %)
Maternal age							
	11–19 years	181	(9.1)	728	(2.4)	1871	(0.7)
	20–24 years	576	(29.1)	5325	(17.8)	27 707	(10.9)
	25–29 years	652	(32.9)	9805	(32.8)	83 106	(32.6)
	30–34 years	393	(19.8)	8341	(27.9)	87 646	(34.4)
	≥35 years	177	(8.9)	5715	(19.1)	54 642	(21.4)
	Missing information	4	(0.2)	0	(0.0)	1	(0.0)
Parity							
	0	717	(36.2)	7988	(26.7)	110 048	(43.2)
	1–2	908	(45.8)	14 497	(48.5)	127 015	(49.8)
	3 or higher	348	(17.6)	7156	(23.9)	10 065	(4.0)
	Missing information	10	(0.5)	273	(0.9)	7845	(3.1)
Education							
	Less than 9 years	465	(23.5)	3779	(12.6)	452	(0.2)
	9 years	334	(16.8)	6453	(21.6)	8734	(3.4)
	12 years	255	(12.9)	7825	(26.2)	95 809	(37.6)
	University or professional education	251	(12.7)	4904	(16.4)	132 919	(52.1)
	Missing information	678	(34.2)	6953	(23.2)	17 059	(6.7)
Self-rated health before pregnancy							
	Very good	93	(4.7)	4469	(14.9)	82 859	(32.5)
	Good	873	(44.0)	17 958	(60.0)	132 152	(51.8)
	Neither good or bad	269	(13.6)	2368	(7.9)	15 139	(5.9)
	Poor	131	(6.6)	804	(2.7)	5023	(2.0)
	Very poor	38	(1.9)	205	(0.7)	1289	(0.5)
	Don’t know	579	(29.2)	4099	(13.7)	18 308	(7.2)
	Missing information	0	(0.0)	11	(0.0)	203	(0.1)
Maternal BMI							
	Underweight (<18.5)	78	(3.9)	899	(3.0)	5424	(2.1)
	Normal weight (18.5–25)	842	(42.5)	12 134	(40.6)	142 808	(56.0)
	Overweight (25–30)	568	(28.6)	9388	(31.4)	57 995	(22.8)
	Obese class I (30–35)	232	(11.7)	4199	(14.0)	21 311	(8.4)
	Obese class II (≥35)	84	(4.2)	1610	(5.4)	9662	(3.8)
	Missing information	179	(9.0)	1684	(5.6)	17 773	(7.0)
Smoking at first antenatal care							
	No	1583	(79.8)	24 968	(83.5)	206 712	(81.1)
	1–9 cigarettes/day	61	(3.1)	567	(1.9)	9604	(3.8)
	10 or more cigarettes/day	13	(0.7)	116	(0.4)	2532	(1.0)
	Missing information	326	(16.4)	4263	(14.3)	36 125	(14.2)
Risky alcohol behaviour at first antenatal care							
	No	1588	(80.1)	24 039	(80.4)	186 729	(73.2)
	Yes	1	(0.1)	43	(0.1)	11 454	(4.5)
	Missing	394	(19.9)	5832	(19.5)	56 790	(22.3)
Gestational age at first antenatal care visit							
	Median (day)	91		63		56	
	Before 14 weeks	1012	(51.0)	23 819	(79.6)	239 135	(93.8)
	14–22 weeks	376	(19.0)	4238	(14.2)	10 384	(4.1)
	After 22 weeks	589	(29.7)	1799	(6.0)	4654	(1.8)
	Missing information	6	(0.3)	58	(0.2)	800	(0.3)

Table 2 presents the combined perinatal health outcomes of refugees, asylum-seekers and undocumented migrants. Migrant women had higher risks of poor self-rated health before pregnancy, gestational diabetes and severe anal sphincter tears than their Swedish counterparts, and their infants had higher risks of preterm birth, low birthweight and low Apgar scores at 5 min. The mean birthweight of migrant term-born infants was 215 g (95% CI 207–222) lower than their Swedish counterparts, whose mean birthweight was 3634 g (95% CI 3632–3636). Migrant women had higher risks of insufficient antenatal care visits, no ultrasound screening, instrumental vaginal or Caesarean section deliveries and missing postpartum visits.

**Table 2:** Proportions of adverse health and health care indicators of migrant women, and Swedish-born women

		Migrant women		Swedish-born women		Migrant women vs. Swedish-born women			
		*N* = 31 897^a^		*N* = 254 973^a^		RR	(95% CI)	RD per 1000 birth	(95% CI)
		%	(95% CI)	%	(95% CI)				
**Maternal health in general**									
Maternal poor self-rated health before pregnancy		18.4	(17.9 to 18.8)	9.7	(9.5 to 9.8)	**1.78**	(1.73 to 1.83)	**75.4**	(70.9 to 80.0)
**Indicators of perinatal health**									
Maternal									
	Pre-eclampsia	1.7	(1.6 to 1.9)	3.2	(3.1 to 3.2)	**0.70**	(0.64 to 0.77)	**–9.1**	(–11.1 to –7.1)
	Gestational diabetes	3.6	(3.4 to 3.8)	1.6	(1.6 to 1.7)	**2.07**	(1.93 to 2.22)	**17.7**	(15.4 to 19.9)
	Severe postpartum haemorrhage (blood loss > 1000 ml)	5.6	(5.3 to 5.9)	7.1	(7.0 to 7.2)	**0.95**	(0.90 to 0.99)	**–3.8**	(–7.1 to –0.5)
	Degree III–IV anal sphincter tear^b^	2.6	(2.4 to 2.8)	2.4	(2.4 to 2.5)	**1.83**	(1.70 to 1.97)	**23.2**	(19.7 to 26.7)
Foetal									
	Stillbirth	0.6	(0.6 to 0.7)	0.3	(0.2 to 0.3)	**2.24**	(1.89 to 2.66)	**3.3**	(2.4 to 4.3)
	Preterm birth	4.4	(4.1 to 4.6)	4.9	(4.8 to 4.9)	**0.92**	(0.87 to 0.97)	**–3.8**	(–6.4 to –1.3)
	Preterm birth (ultrasound estimation)	4.3	(4.0 to 4.5)	4.8	(4.7 to 4.9)	**0.91**	(0.86 to 0.97)	**–4.2**	(–6.8 to –1.7)
	Small for gestational age	3.7	(3.5 to 3.9)	1.8	( .8 to 1.9)	**2.58**	(2.42 to 2.76)	**28.4**	(25.2 to 31.2)
	Birth weight < 2500 g	3.7	(3.5 to 3.9)	2.9	(2.9 to 3.0)	**1.46**	(1.36 to 1.55)	**12.8**	(10.2 to 15.3)
	Apgar score < 7 at 5 min	2.2	(2.0 to 2.3)	1.4	(1.3 to 1.4)	**1.77**	(1.62 to 1.93)	**10.2**	(8.2 to 12.1)
Mean birth weight of term birth (grams)		3, 469	(3463 to 3475)	3, 634	(3632 to 3636)	**–215** (–222 to –207)			
**Indicators of perinatal health care**									
Antenatal care usage									
	Inadequate antenatal care visits	7.3	( 7.0 to 7.6)	6.6	( 6.5 to6.7)	**1.80**	(1.71 to 1.98)	**28.8**	(25.8 to 31.9)
	No ultrasound screening	1·0	( 0.9 to 1.1)	0·4	( 0.3 to 0.4)	**3.40**	( 2.93 to 3.95)	**7.0**	( 5.7 to 8.3)
Use of professional translator in antenatal care		47.2	(46.6 to 47.8)	1.0	(1.0 to 1.1)	**1.86**	(1.81 to 1.91)	**387.2**	(365.8 to 408.6)
Abnormal CTG at arriving delivery ward		5.2	(5.0 to 5.5)	3.9	(3.9 to 4.0)	0.88	(0.70 to 1.10)	–6.3	(–16.8 to 4.3)
Mode of delivery									
	Non-instrumental vaginal	77.6	(77.1 to 78.0)	78.2	(78.0 to 78.3)	**0.90**	(0.89 to 0.91)	**–77.7**	(–83.6 to –71.9)
	Instrumental vaginal	5.0	(4.7 to 5.2)	5.7	(5.6 to 5.8)	**1.42**	(1.35 to 1.49)	**22.7**	(19.0 to 26.5)
	Planned Caesarean section	7.3	(7.0 to 7.6)	6.9	(6.8 to 7.0)	**1.12**	(1.07 to 1.17)	**8.3**	(5.0 to 11.6)
	Unplanned Caesarean section	10.1	(9.8 to 10.5)	9.3	(9.2 to 9.4)	**1.53**	(1.47 to 1.58)	**46.7**	(42.1 to 51.3)
Postpartum care usage									
	Missing postpartum visit	39.6	(39.0 to 40.1)	24.9	(24.8 to 25.1)	**1.40**	(1.38 to 1.43)	**103.9**	(98.0 to 109.7)

All models adjusted for maternal age and parity in quadratic forms. Bold values indicate *P* < 0.05.

^a^Excluded women with missing data on maternal age or parity.

^b^Excluded all births delivered through Caesarean section.

Migrant and Swedish women had similar risks of having abnormal CTG tracing at admission to the delivery ward. Maternal diagnoses of pre-eclampsia, preterm birth and severe postpartum haemorrhage were less common in migrant women than Swedish women. We cannot make statistical inference on maternal death, due to its rarity (data not shown).

Table 3 compares asylum-seeking and undocumented migrant women to refugee women. Asylum-seeking and undocumented migrant women without residency had a higher risk of poor self-rated health before pregnancy with a RR of 1.84 (95% CI 1.72–1.97) and RD of 145.5 (95% CI 124.9–166.2) per 1000 births. The rates were also higher for preterm birth (RR 1.47, 95% CI 1.21–1.79 and RD 19.3, 95% CI 7.6–13.0 per 1000 births), and low birthweight (RR 1.36, 95% CI 1.11–1.66 and RD 15.9, 95% CI 3.9–28.0 per 1000 births). Mean birthweight of term-born babies was borderline lower among asylum-seeker and undocumented migrants (beta –25, 95% CI –53 to 4) than refugee women. Asylum-seeking and undocumented migrant women were also more likely to have planned Caesarean sections than refugee women (RR 1.34, 95% CI 1.15–1.55 and RD 24.2, 95% CI 10.2–38.3). They also had higher risks of inadequate antenatal care, having no ultrasound screenings, and missing postpartum care visits.

**Table 3:** Adjusted RRs and RDs of indicators of health and health care in asylum-seeking and undocumented migrant women and refugee women (*N* = 31 609^a^)

		Asylum-seekers and undocumented migrants		Refugee women		Asylum-seekers and undocumented migrants vs. refugee women			
		*N* = 1969		*N* = 29 640		RR	(95% CI)	RD per 1000 births	(95% CI)
		*n*	(%)	*n*	(%)				
**Maternal health in general**									
Maternal poor self-rated health before pregnancy		741	(37.6)	5059	(17.1)	**1.84**	(1.72 to 1.97)	**145.5**	(124.9 to 166.2)
**Indicators of perinatal health**									
Maternal									
	Pre-eclampsia	34	(1.7)	510	(1.7)	1.19	(0.82 to 1.73)	3.2	(–4.2 to 10.7)
	Gestational diabetes	44	(2.2)	1096	(3.7)	0.76	(0.56 to 1.03)	**–9.0**	(–17.7 to –0.4)
	Severe postpartum haemorrhage (blood loss > 1000 ml)	82	(4.2)	1693	(5.8)	**0.78**	(0.62 to 0.98)	**–12.7**	(–23.2 to –2.2)
	Degree III–IV anal sphincter tear^b^	52	(2.6)	766	(3.1)	0.94	(0.71 to 1.25)	–1.9	(–10.4 to 6.6)
Foetal									
	Stillbirth	14	(0.7)	192	(0.7)	1.30	(0.74 to 2.29)	2.0	(–2.8 to 6.7)
	Preterm birth	121	(6.2)	1227	(4.1)	**1.47**	(1.21 to 1.79)	**19.3**	(7.6 to 31.0)
	Preterm birth (ultrasound estimation)	101	(6.1)	1152	(4.1)	**1.48**	(1.19 to 1.83)	**19.3**	(6.8 to 31.8)
	Small for gestational age	88	(4.5)	1063	(3.6)	1.20	(0.95 to 1.51)	7.1	(–2.9 to 17.1)
	Birth weight < 2500 g	94	(4.8)	1039	(3.5)	**1.36**	(1.11 to 1.66)	**15.9**	(3.9 to 28.0)
	Apgar score < 7 at 5 min	41	(2.1)	625	(2.1)	1.13	(0.81 to 1.58)	2.7	(–5.2 to 10.6)
Mean birth weight of term birth (g)		3420	(3394 to 3446)	3472	(3466 to 3479)	–25 (–53 to 4)			
**Indicators of perinatal health care**									
Antenatal care usage									
	Inadequate antenatal care visits	313	(15.9)	1729	(5.8)	**2.56**	(2.27 to 2.89)	**91.5**	( 74.8 to 108.3)
	No ultrasound screening	79	(4.0)	202	(0.7)	**5.43**	(4.02 to 7.10)	**29.8**	( 20.7 to 38.8)
Use of professional translator in antenatal care		1606	(81.6)	11 651	(39.3)	**1.70**	(1.67 to 1.74)	**359.9**	(341.6 to 378.2)
Abnormal CTG at arriving delivery ward		88	(4.5)	1558	(5.3)	0.88	(0.70 to 1.10)	–6.3	(–16.8 to 4.3)
Mode of delivery									
	Non-instrumental vaginal	1463	(74.3)	23 072	(77.8)	**0.96**	(0.93 to 0.98)	**–33.8**	(–54.6 to –13.0)
	Instrumental vaginal	112	(5.7)	1455	(4.9)	1.03	(0.84 to 1.26)	1.4	(–8.8 to 11.6)
	Planned Caesarean section	185	(9.4)	2135	(7.2)	**1.34**	(1.15 to 1.55)	**24.2**	(10.2 to 38.3)
	Unplanned Caesarean section	209	(10.6)	2979	(10.1)	1.08	(0.94 to 1.24)	8.1	(–7.0 to 23.3)
Postpartum care usage									
	Missing postpartum visit	812	(41.2)	11 711	(39.5)	**1.15**	(1.09 to 1.21)	**59.6**	(36.0 to 83.3)

All models were adjusted for maternal education, age and parity in quadratic forms, calendar year of delivery and mother’s country of origin. Models of maternal and foetal outcomes, abnormal CTG on arrival at the delivery ward and mode of delivery were also adjusted for maternal BMI categories and height in continuous form. Bold values indicate *P* < 0.05.

^a^Excluded women with missing data on maternal age or parity.

^b^Excluded all births delivered by Caesarean section.

Refugees and asylum-seekers, including undocumented migrants, had similar risk of pre-eclampsia, gestational diabetes and III–IV degree anal sphincter tears. However, asylum-seeking and undocumented migrant women were less likely to have a severe postpartum haemorrhage (RR 0.78, 95% CI 0.62–0.98 and RD –12.7, 95% CI –23.2 to –2.2 per 1000 births). A sensitivity analysis of Syrian women showed similar findings for maternal and infant outcomes among asylum-seekers and undocumented migrants compared to refugees (data not shown).

Supplementary table S1 presents an analysis of the excluded from the study population due to missing information on their country of birth and personal identification numbers (*N* = 675). They show similar risks of adverse birth outcomes as the asylum-seekers and undocumented migrants included in the study population.

## Discussion

This study shows that from 2014 to 2017 pregnant refugee women in Sweden had higher risks of poor self-rated health, gestational diabetes and severe anal sphincter tears, as well as higher risks of adverse infant outcomes including stillbirth, low birthweight, small for gestational age and low Apgar score than Swedish-born women. Asylum-seeking and undocumented migrant women were particularly vulnerable, with higher risks of poor maternal self-rated health, preterm birth and missed perinatal visits than refugee women with established residency.

Our results suggest that legal residency status had a potent influence on the perinatal health of refugee women and their infants. Previous studies have demonstrated the connection between maternal psychosocial stress and preterm birth.^23–25^ Given higher rates of poor self-rated health of asylum-seeking and undocumented migrant women, we suspect that the stress of uncertain immigration status is linked to the increase in preterm birth in the study population. The negative impact of stress related to uncertain asylum procedures has been shown to increase over time, manifesting in increased symptom loads and changed cortisol metabolism.^26^

Pregnant refugee and asylum-seeking women, including undocumented migrants, in European countries have the greatest needs for special attention and care from maternal health services. The International Organization for Migration (IOM) surveys conducted in 2017 demonstrated alarmingly high rates of rape and sexual exploitation among asylum-seeking women en route to Europe.^4^ In our study, asylum-seeking and undocumented migrant women were at the highest risk for negative outcomes; yet many EU member states provide only limited maternity and reproductive health care for asylum-seekers and undocumented migrants, effectively ignoring human rights conventions and breaching common EU policy, with far-reaching consequences for these women and their unborn children.^27^

However, our study also indicates that equal rights to maternity care, which exists in Sweden, is not sufficient to guarantee equal access to care. Missed antenatal visits and ultrasound screening may be partially due to a later arrival in the destination country (Sweden); however, the increased risk of missing postnatal care visits clearly indicates lower care usage and missed opportunities to improve maternity care. There may also be barriers to the delivery of maternity care, including language and more subtle barriers related to cultural differences in belief systems and being less familiar with preventative care. Medically trained interpreters and cultural mediators can facilitate communication between midwives, society and refugee women.^28^^,^^29^ The literature suggests a number of factors that could improve the quality and uptake of maternity care by refugee and asylum-seeking women, such as more stable housing and continuous information flow between different healthcare organizations.^28^^,^^30^ Ensuring continuity of care, educating newly resettled refugees and asylum-seekers about perinatal care and providing health professionals with cultural sensitivity training have been recommended to meet their special needs.^31^^,^^32^ Policies and regulations that endorse human-rights–based approaches to maternal and infant health need to be implemented,^33^^,^^34^ in order to target the social determinants that affect women’s rights and access to services, such as lack of legal status, as illustrated in this study.

Pre-eclampsia, preterm birth and severe postpartum haemorrhage were less common in migrant women than in Swedish women. The result on pre-eclampsia is consistent with a previous Swedish study and a systematic review of pregnancy-related hypertensive disorder in migrant populations.^8^^,^^35^ A Swedish study also showed lower risks of preterm birth among Middle Eastern, Somali and Ethiopian/Eritrean migrants.^36^ The multifaceted mechanisms of preterm birth and pre-eclampsia will require further investigation to explain these findings. Lower birthweight and possibly smaller placental size of babies with migrant mothers may partly explain migrants’ lower risk of postpartum haemorrhage than the Swedish population.^37^^,^^38^

The Swedish Pregnancy Register provided a unique opportunity to investigate the perinatal health of refugee women in Sweden, where free health care regardless of residency status is guaranteed. To the authors’ knowledge, this was the first population-based study to compare the impact of legal residency on perinatal health by comparing refugees with asylum-seekers and undocumented migrants. The findings may not be directly generalized to other contexts and setting, but provide valuable information for future policy-making in response to forced migration.

Duration of residence could not be included in the analysis, given the unknown date of arriving in Sweden. Comparison of asylum-seekers and refugees from Syria (who migrated more recently) showed results consistent with those of the main analysis. Longer duration of residency may also be associated with higher risk of preterm birth.^39^ Thus, it is unlikely that the observed differences between migrant populations were confounded by duration of residence.

## Conclusion

This Swedish study showed that asylum-seeking and undocumented migrant women had poorer self-rated health before giving birth than refugee women, and also received less perinatal maternity care. They also had a higher risk of preterm birth than refugee women. More effective asylum procedures and greater intervention by health care providers, in partnership with other public service sectors, are critical for improving the pregnancy outcomes of forcibly displaced and newly resettled women.

## Supplementary Material

ckz120_Supplementary_Data
