# Supplementary material for: Perinatal health of refugee and asylum-seeking women in Sweden 2014–17: a register-based cohort study
Source: Eur J Public Health. 2019 Jul 4;29(6):1048–55. doi: 10.1093/eurpub/ckz120 (PMC6896976; doi:10.1093/eurpub/ckz120)
Supplement: ckz120_Supplementary_Data [file ckz120_supplementary_data.docx]

**Table S1. Birth outcomes of women without antenatal care record and personal identification number**

|  | Women without antenatal care and personal identification number | |
| --- | --- | --- |
|  | N=7356 | |
|  | n | % |
| Abnormal CTG at arriving delivery ward | 387 | 5.26 |
| Stillbirth | 44 | 0.60 |
| Preterm birth | 508 | 6.91 |
| Birthweight<2500g | 402 | 5.46 |
| Apgar score<7 at 5 minutes | 179 | 2.48 |
